# *Cacna1c* deficiency in parvalbumin-expressing neurons promotes anxiety and passive stress-coping behavior

**DOI:** 10.1038/s41598-026-48841-4

**Published:** 2026-04-20

**Authors:** Srivaishnavi Loganathan, Chen Zhao, Jan M. Deussing

**Affiliations:** 1 Research Group Molecular Neurogenetics, Max Planck Institute of Psychiatry, Kraepelinstr. 2-10, 80804 Munich, Germany; 2 International Max Planck Research School for Translational Psychiatry (IMPRS-TP), Munich, Germany

**Keywords:** Cacna1c, Ca_v_1.2, Parvalbumin, Glutamatergic, Anxiety, Stress, Mouse model, Neuroscience, Physiology

## Abstract

The cross-disorder risk gene *CACNA1C* encoding the α1 subunit of the L-type calcium channel Ca_v_1.2 has repeatedly been associated with various psychiatric disorders. *Cacna1c* is expressed in parvalbumin positive (PV^+^) neurons which play an important role in regulating excitation-inhibition (E/I) balance and their dysregulation has been implicated in different psychiatric conditions. To address the cell type-specific contribution of Ca_v_1.2 to endophenotypes related to psychiatric disorders, we generated mice with a conditional *Cacna1c* deletion in PV^+^ neurons (Ca_v_1.2-PV). Male knockout mice exhibited increased anxiety-like behavior, and a more passive stress-coping strategy in the forced swim test, while locomotion, social behavior, and cognitive performance were unaffected. In contrast, mice lacking Ca_v_1.2 in forebrain glutamatergic neurons (Ca_v_1.2-Nex) displayed enhanced active stress-coping, revealing a bidirectional, cell type-specific role of Ca_v_1.2 in stress-induced behaviors. cFos mapping following stress exposure identified distinct activation patterns in stress-responsive brain regions, suggesting distinct circuit mechanisms underlying these opposing behavioral phenotypes. Together, these findings identify Ca_v_1.2 in PV^+^ neurons as a critical regulator of anxiety and stress-coping behavior and highlight complementary contributions of inhibitory and excitatory circuits to stress adaptation, with implications for targeted therapeutic strategies in psychiatric disorders.

**Supplementary Information:**

The online version contains supplementary material available at 10.1038/s41598-026-48841-4.

## Introduction

Parvalbumin-expressing (PV^+^) neurons, in particular GABAergic interneurons, are integral to maintaining the excitation-inhibition (E/I) balance in cortical circuits through inhibition of neighboring pyramidal neurons^1,2^. These fast-spiking neurons are characterized by their ability to regulate synchronized neuronal activity, particularly through the generation of gamma oscillations, which are essential for higher-order cognitive processes such as sensory integration, working memory and attention^3–5^. Dysregulation of PV^+^ neuron activity has been implicated in several neurological and psychiatric conditions, including epilepsy, schizophrenia (SCZ), major depressive disorder (MDD), autism spectrum disorders (ASD) and Alzheimer’s disease^2,6,7^. Studies in mouse models of autism, such as those related to the Fragile X syndrome, revealed deficits in PV^+^ neuron excitability and synaptic output, leading to hyperexcitation of cortical networks^8,9^. In this context, the absence of contactin-associated protein-like 2, another autism-associated gene, has been demonstrated to lead to abnormal neuronal migration and network activity along with reduced numbers of PV^+^ neurons resulting in disease-associated behavioral abnormalities^10^. Similarly, several mouse models of disease-associated risk genes have highlighted the importance of PV^+^ neurons in the pathogenesis of psychiatric conditions. For example, conditional deletion of NMDA receptor subunit NR1 in PV^+^ neurons disrupts gamma oscillations and impairs working memory, a mechanism relevant to SCZ^11^. Mice with a conditional deletion of the receptor tyrosine-protein kinase ErbB4, a protein selectively expressed in PV^+^ interneurons and a risk factor for SCZ, replicate aspects of disease phenotypes^12–14^. In mouse models of mood disorders, chronic stress increases PV expression and alters inhibitory signaling, particularly in the prefrontal cortex and hippocampus, leading to disrupted emotion-related behavioral responses^15,16^.

Calcium signaling plays a fundamental role in neuronal function, regulating processes such as neurotransmitter release, synaptic plasticity, and gene expression^17^. Among the calcium channels that mediate this signaling, L-type voltage-gated calcium channels (L-VGCCs), particularly Ca_v_1.2, has garnered significant attention due to its contribution to long-term changes in neuronal activity and its implications in neurological and psychiatric disorders^18–20^. *CACNA1C*, encoding the α1 subunit of Ca_v_1.2, is one of the most robust and consistently replicated genetic risk factors for multiple psychiatric disorders including SCZ, bipolar disorder, MDD, ASD and attention-deficit hyperactivity disorder^21^. Ca_v_1.2 is characterized by high-voltage activation and sustained calcium influx, thereby driving activity-dependent processes such as excitation-transcription coupling and expression of calcium-dependent genes^22,23^. This channel is predominantly localized postsynaptically at dendrites and soma, and plays a vital role in regulation of synaptic plasticity, dendritic development, learning and memory formation^17,18,24–27^.

While Ca_v_1.2 has been extensively investigated in several neuronal and glial cell types, its functional role in inhibitory neurons, particularly PV^+^ neurons, remains underexplored. Emerging evidence suggests that Ca_v_1.2 channels are expressed in PV^+^ neurons and may influence their maturation as well as electrophysiological properties, synaptic connectivity, and network activity^28–30^. However, the specific role of Ca_v_1.2 in PV^+^ neurons and its contribution to phenotypes related to psychiatric disorders has not been investigated. To study the role of Ca_v_1.2 in PV^+^ neurons, we used a conditional knockout mouse model combining a PV^+^ neuron-specific Cre driver with mice harboring a floxed *Cacna1c* allele. A Cre-dependent disruption of *Cacna1c* leads to the selective inactivation of Ca_v_1.2 in all PV^+^ neurons unraveling its specific role in modulating emotional behaviors and the response to acute stress. Depending on whether Ca_v_1.2 is inactivated in inhibitory PV^+^ or excitatory glutamatergic neurons, stress differentially recruits neural circuits as indicated by the differential expression of the immediate early gene cFos in stress responsive brain areas. This study provides novel insights into the role of Ca_v_1.2 in PV^+^ neurons modulating the stress response and provides entry points for further exploration of the underlying mechanisms.

## Results

### Ca_v_1.2 inactivation in parvalbumin-expressing neurons

**Fig. 1:**
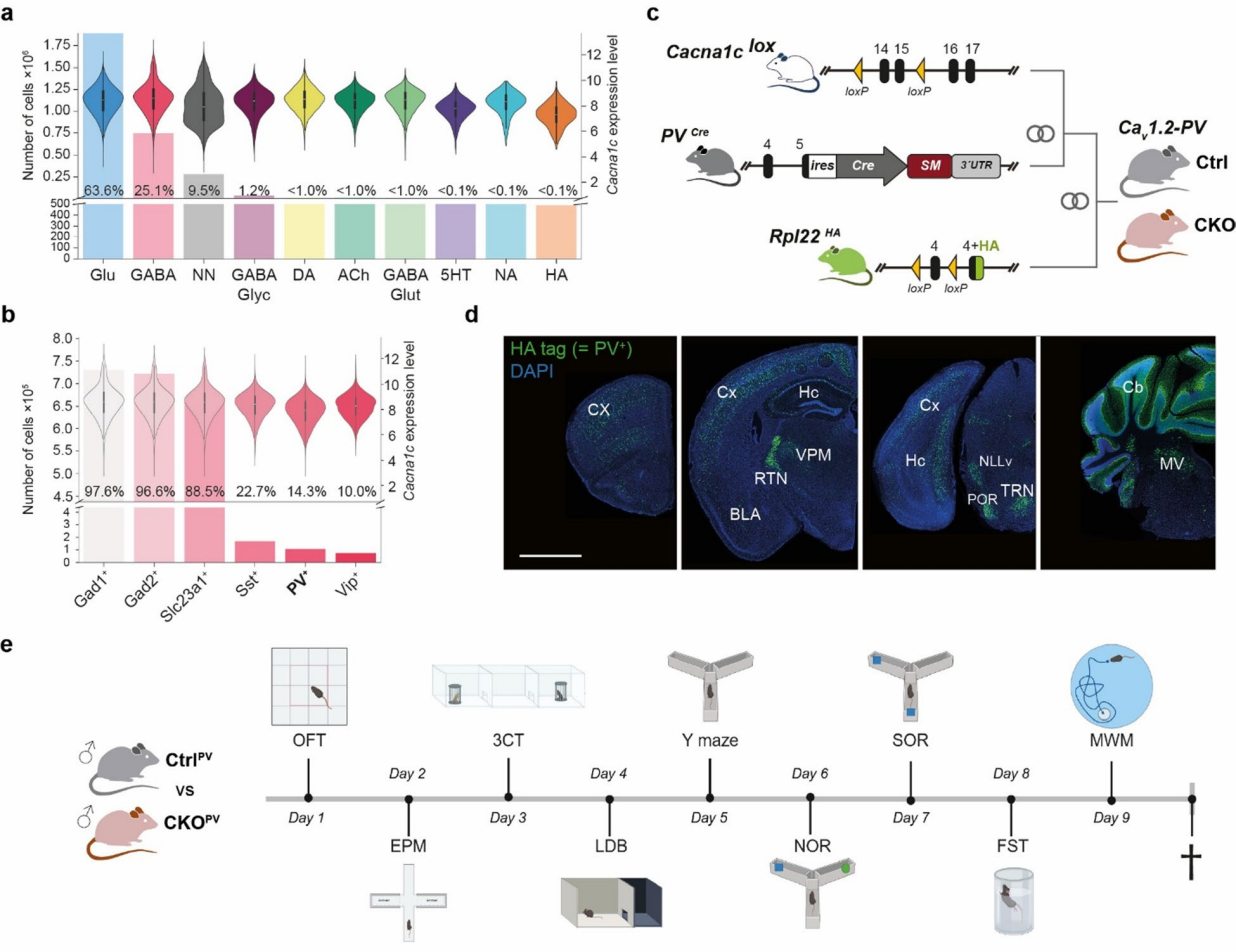
Analysis of *Cacna1c* expression and conditional inactivation in PV^+^ neurons. **a** *Cacna1c* expression across neurotransmitter-defined cell groups and **b** GABAergic subpopulations. Abbreviations: NN, non-neuronal. **c** Scheme depicting the strategy for generation of the conditional Ca_v_1.2-PV knockout mouse line. Abbreviations: SM, selection marker. **d** Analysis of Cre recombination pattern by antibody staining against HA tag of RPL22-HA. Abbreviations: BLA, basolateral amygdala; Cb, cerebellum; Cx, cortex; Hc, hippocampus; MV, medial vestibular nucleus; NLLv, nucleus of the lateral lemniscus ventral, ventral part; POR, superior olivary complex, periolivary region; RTN, reticular thalamic nucleus; TRN, tegmental reticular nucleus; VPM, ventral posteromedial nucleus of the thalamus. **e** Timeline depicting behavioral test battery (created with BioRender.com/tye3bkd).

*Cacna1c*, the α1 subunit of the L-type calcium channel Ca_v_1.2, is widely expressed throughout different neuronal subtypes in the murine brain. Analysis of single-cell RNA-sequencing data confirmed its presence in the majority of glutamatergic (63,6%) and GABAergic (25.1%) neurons (Fig. 1a). Among different GABAergic subpopulations, *Cacna1c*-expressing PV^+^ neurons represent 14,3% of all GABAergic neurons (Fig. 1b). To investigate the role of Ca_v_1.2 in this neuronal subpopulation, we bred floxed *Cacna1c* mice (*Cacna1c*^*lox*^) to a PV-Cre driver line generating a conditional knockout mouse model with PV^+^ neuron-specific Ca_v_1.2 inactivation (Ca_v_1.2-PV). To overcome the unavailability of CACNA1C-specific antibodies suitable for immunohistochemistry, we included the Cre-dependent *Rpl22*^*HA*^ (RiboTag) allele to readily monitor the efficacy and spatial pattern of recombination by immunostaining for the HA-tagged RPL22 variant activated by Cre-mediated recombination (Fig. 1c). Immunofluorescence staining of brain sections from conditional knockout mice harboring the Cre recombinase (CKO^PV^) revealed HA-tag positive neurons throughout the brain including the cortex, hippocampus, thalamus, amygdala, and cerebellum in agreement with endogenous PV expression (Fig. 1d). In contrast, RPL22-HA expressing cells were undetectable in Cre negative control mice (Ctrl^PV^) (data not shown). Taken together, the immunofluorescence staining confirmed the efficient activation of the reporter which serves as a proxy for the successful inactivation of *Cacna1c* and thus Ca_v_1.2 in PV^+^ neurons. Subsequently, CKO^PV^ mice and Ctrl^PV^ littermates were subjected to a behavioral test battery assessing locomotion, anxiety-related and social behavior, learning and memory as well as stress-coping behavior (Fig. 1e).

### Ca_v_1.2 deficiency in PV^+^ neurons increases anxiety-related behavior

**Fig. 2:**
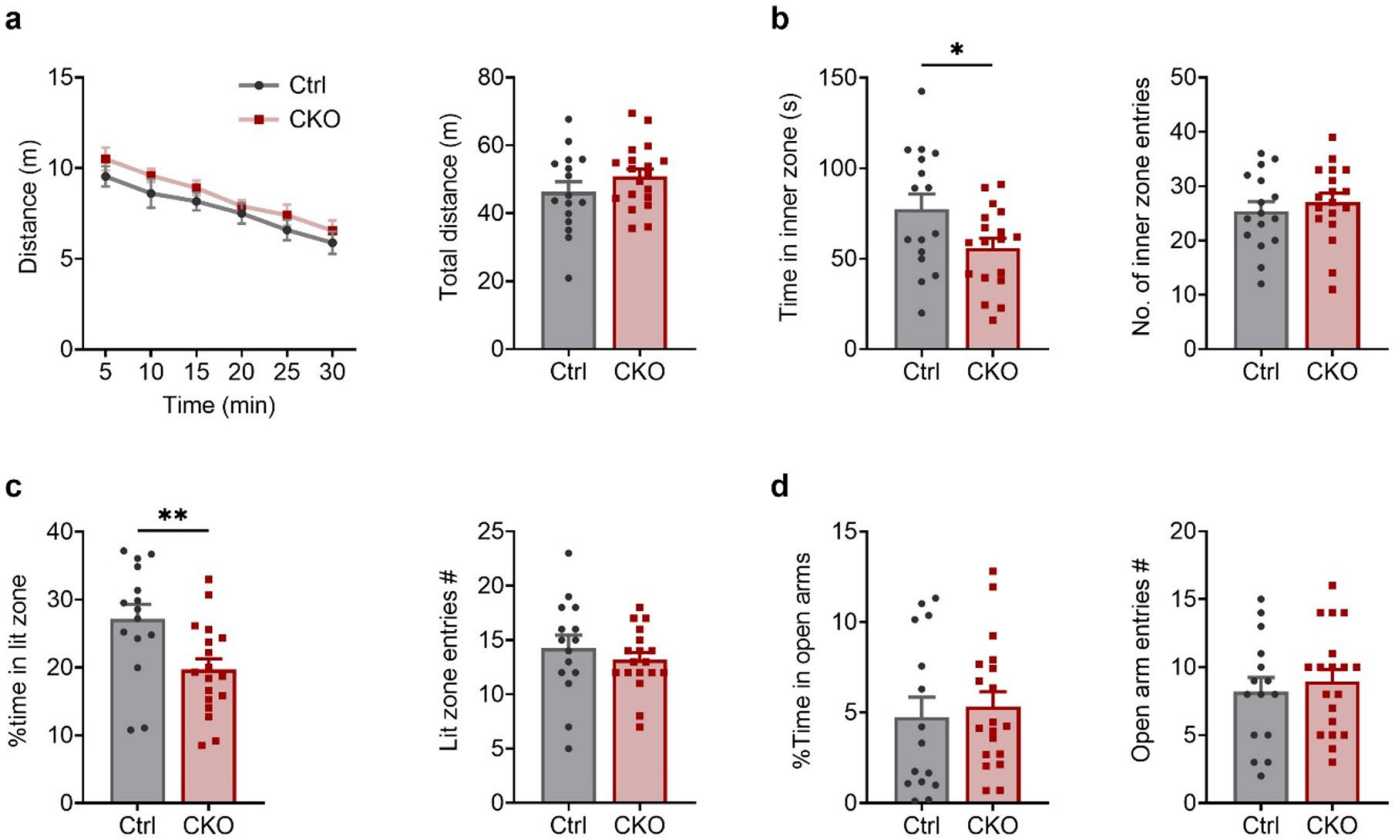
Loss of Ca_v_1.2 in PV^+^ neurons promotes anxiety-related behavior. **a** Distance travelled in 5-min segments across 30-min duration and total distance travelled during OFT. **b** Time spent in and number of entries into the inner zone of OF arena. **c** Percentage time spent in and number of entries into the lit zone of LDB. **d** Percentage time spent in and number of entries into the open arms of elevated plus maze. The p values are from student´s t test, ^*^p < 0.05, ^**^p < 0.01, data are presented as mean ± S.E.M.(Ctrl^PV^: *n* = 16, CKO^PV^: *n* = 19).

To determine the impact of Ca_v_1.2 deletion in PV^+^ neurons on locomotor activity and anxiety-related behavior, male Ca_v_1.2-PV mice were tested in the open field test (OFT), light-dark box (LDB) and elevated plus maze (EPM). In the OFT, CKO^PV^ mice and their Ctrl^PV^ littermates displayed comparable exploratory behavior, as indicated by similar locomotor activity and habituation across time (two-way ANOVA-RM; interaction, F_(5, 165)_ = 0.1605, *p* = 0.9765; genotype, F_(1, 33)_ = 1.617, *p* = 0.2124, time, F_(5, 165)_ = 26.99, *p* < 0.0001) as well as with respect to the total distance traveled (Student’s t test, t_33_ = 1.272, *p* = 0.2124) (Fig. 2a). Despite normal locomotion, CKO^PV^ mice exhibited increased anxiety-related behavior in the OFT, spending significantly less time in the inner zone compared to Ctrl^PV^ mice (Student’s t test: t_32_ = 2.202, *p* = 0.0350), while the number of entries into the inner zone remained similar (Student’s t test: t_32_ = 0.6697, *p* = 0.5078) (Fig. 2b). In the LDB, CKO mice also spent significantly less time in the lit zone (Student’s t test, t_31_ = 2.858, *p* = 0.0076), although the number of entries into the lit zone was comparable between the groups (Student’s t test, t_31_ = 0.8376, *p* = 0.4087) (Fig. 2c). Interestingly, Ctrl^PV^ and CKO^PV^ mice were indistinguishable in the EPM, displaying comparable open arm exploration (% open arm time: Student’s t test, t_32_ = 0.4450, *p* = 0.6593, open arm entries: Student’s t test, t_32_ = 0.5556, *p* = 0.5823) (Fig. 2d). Together, these findings indicate that Ca_v_1.2 deficiency in PV^+^ neurons promotes increased anxiety-related behavior as observed in the OFT and LDB, without altering basal locomotion.

### Ca_v_1.2 deficiency in PV^+^ neurons does not alter social behavior or cognitive abilities

**Fig. 3:**
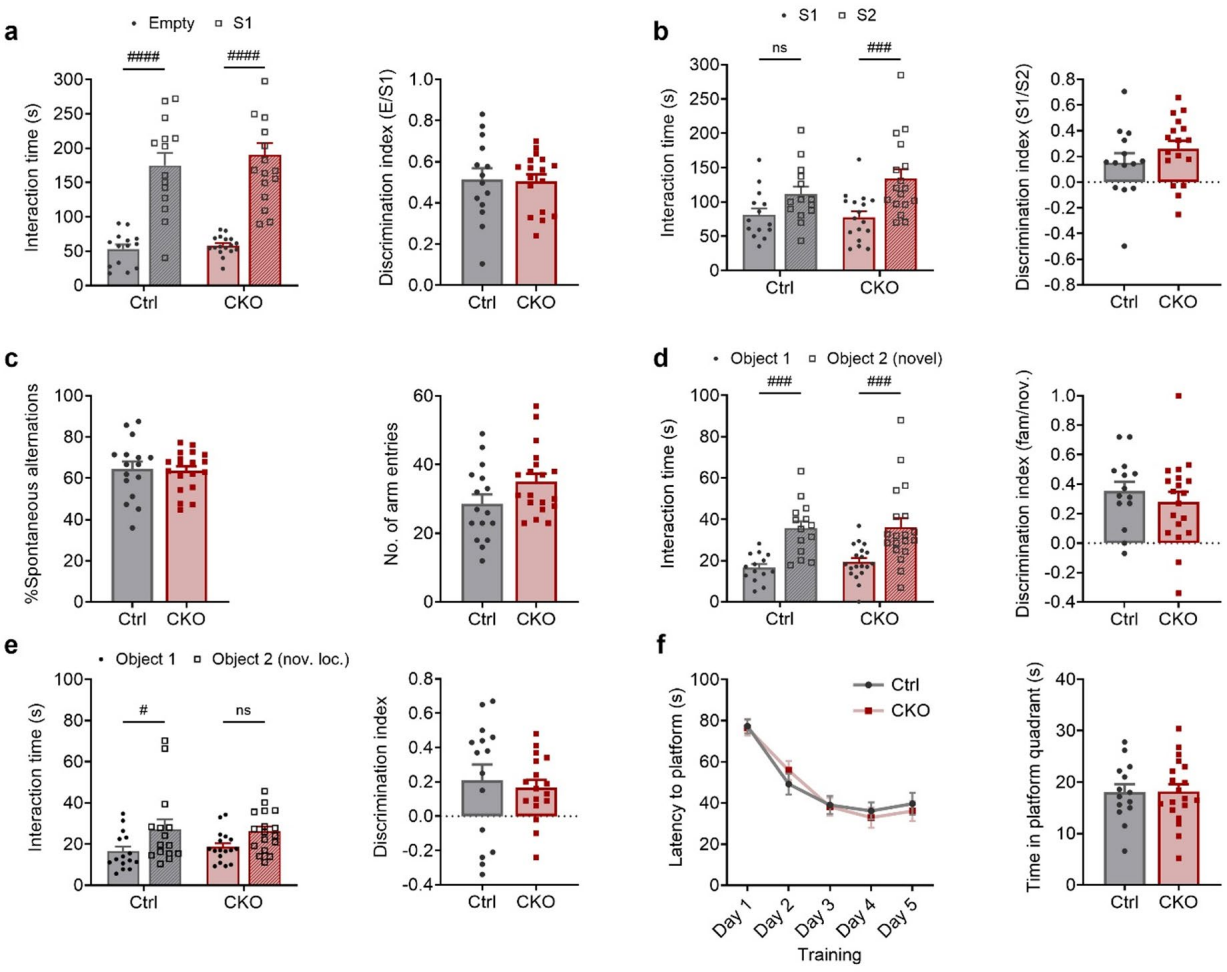
Ca_v_1.2 deficiency in PV^+^ neurons does not alter social behavior or cognitive performance. **a** Interaction times and discrimination indices of Ctrl^PV^ and CKO^PV^ mice during the social preference phase of 3CT. **b** Interaction times and discrimination indices of Ctrl^PV^ and CKO^PV^ mice during the social novelty phase of 3CT. **c** Percentage spontaneous alternations and number of arm entries in Y-maze. **d** Interaction times and discrimination indices of Ctrl^PV^ and CKO^PV^ mice in novelty preference phase of NOR. **e** Interaction times and discrimination indices of Ctrl^PV^ and CKO^PV^ mice in novelty preference phase of SOR. **f** Latency to reach hidden platform during training phase of MWM and time spent in the platform quadrant during probe test of MWM. The p values are from two-way ANOVAs with Bonferroni *post hoc* test, ^#^*p* < 0.05, ^###^*p* < 0.001, ^####^*p* < 0.0001, data are presented as mean ± S.E.M. (Ctrl^PV^: *n* = 16, CKO^PV^: *n* = 19).

Dysfunction of PV^+^ neurons has been linked to abnormal social behavior, particularly in ASD mouse models^31^. Therefore, we assessed Ca_v_1.2-PV mice using the classical three-chambered social interaction test (3CT). In the social preference trial, both Ctrl^PV^ and CKO^PV^ animals interacted significantly more with the social mouse (S1) than with the empty holder (two-way ANOVA: interaction, F_(1, 58)_ = 0.1511, *p* = 0.6989; genotype, F_(1, 58)_ = 0.6155, *p* = 0.4359; social mouse S1, F_(1, 58)_ = 94.06, *p* < 0.0001), with no genotype differences in the discrimination indices (Student’s t test, t_29_ = 0.1449, *p* = 0.8858) (Fig. 3a). Similarly, in the social novelty trial, both groups preferred the novel social mouse (S2) over the familiar one (S1) (two-way ANOVA: interaction, F_(1, 58)_ = 1.360, *p* = 0.2484; genotype, F_(1, 58)_ = 0.7449, *p* = 0.3916; social mouse S2, F_(1, 58)_ = 14.92, *p* = 0.0003) with comparable discrimination indices (Student’s t test, t_29_ = 1.141, *p* = 0.2632) (Fig. 3b).

We next assessed cognitive performance in learning and memory tasks including the Y-maze, novel object recognition (NOR), spatial object recognition (SOR) and Morris water maze (MWM). In the Y-maze, both Ctrl^PV^ and CKO^PV^ animals showed similar spontaneous alternations and arm entries indicating unaltered working memory (Student’s t test, %spontaneous alternations: t_33_ = 0.2116, *p* = 0.8337; arm entry: t_33_ = 1.831, *p* = 0.0761) (Fig. 3c). In the retrieval phase of the NOR test, both groups demonstrated similar discrimination abilities, spending more time interacting with the novel object vs. familiar object (two-way ANOVA, interaction: F_(1,62)_ = 0.1018, *p* = 0.7507; genotype: F_(1,62)_ = 0.2879, *p* = 0.5935, object: F_(1,62)_ = 30.37, *p* < 0.0001; discrimination index: Student’s t test, t_31_ = 0.7544, *p* = 0.4563) (Fig. 3d). In the retrieval phase of the SOR, both groups showed comparable discrimination abilities, even though CKO^PV^ mice spent similar time interacting with objects in familiar and novel location (two-way ANOVA, interaction: F_(1,60)_ = 0.2794, *p* = 0.5990; genotype: F_(1,60)_ = 0.03701, *p* = 0.8481, object: F_(1,60)_ = 9.787, *p* = 0.0027; discrimination index: Student’s t test, t_30_ = 0.4519, *p* = 0.6546) (Fig. 3e).

In the MWM spatial memory test, Ctrl^PV^ and CKO^PV^ mice exhibited identical learning curves during training, with similar latencies to find the hidden platform (two-way ANOVA-RM: interaction, F_(4, 128)_ = 0.5211, *p* = 0.7204; genotype, F_(1, 32)_ = 0.007799, *p* = 0.9302; time, F_(4, 128)_ = 37.93, *p* < 0.0001) (Fig. 3f). Likewise, no differences were observed during the probe test, as both groups spent equal time in the target quadrant (Student’s t test, t_31_ = 0.04488, *p* = 0.9645) (Fig. 3f). Collectively, these findings indicate that Ca_v_1.2 deficiency in PV^+^ neurons does not impair social behavior or working and spatial memory.

### Cell type-specific Ca_v_1.2 deletion reveals bidirectional regulation of stress-coping behavior and associated neural activity

**Fig. 4:**
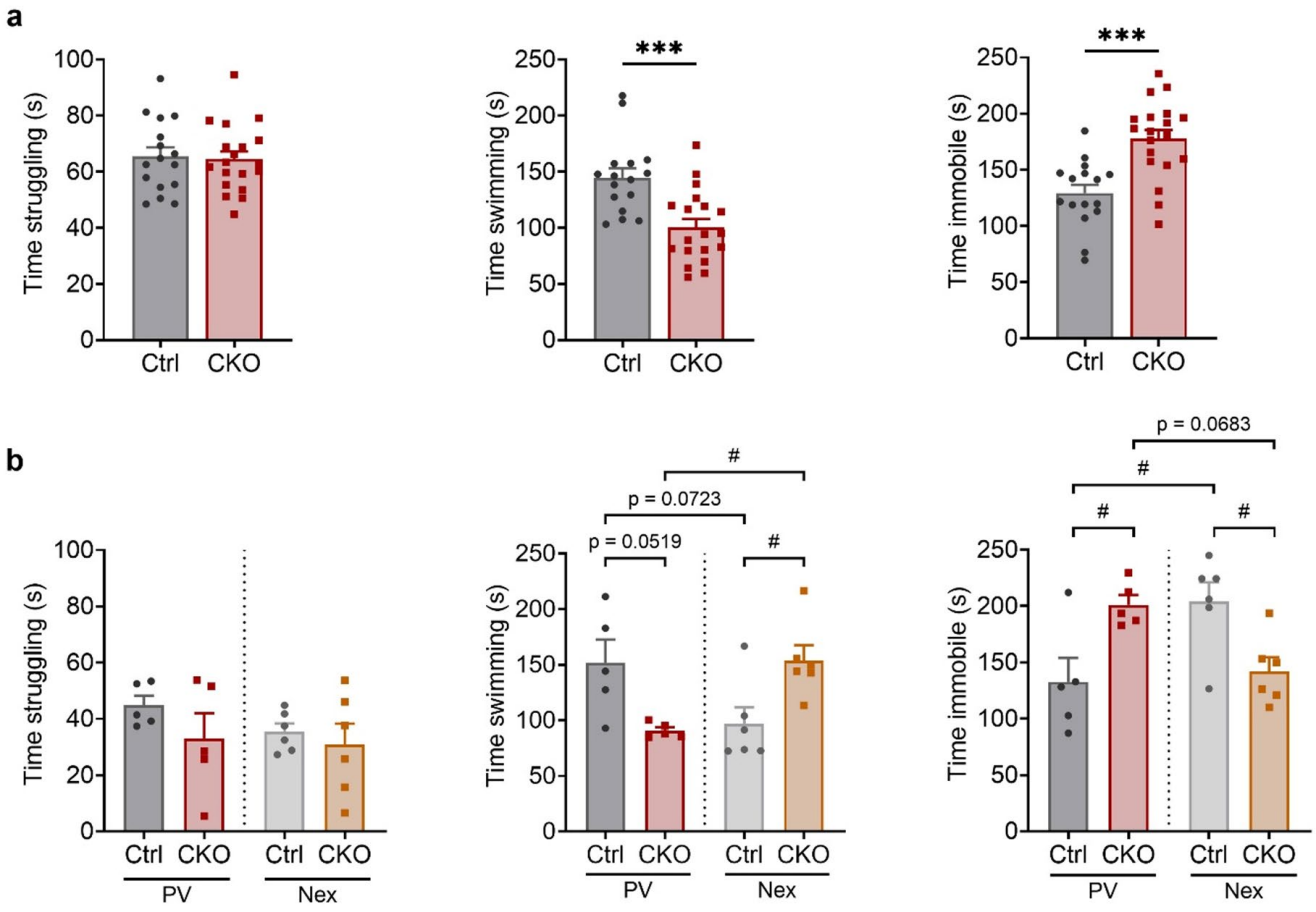
Ca_v_1.2 deficiency in PV^+^ neurons facilitates passive stress-coping behavior. **a** Time Ca_v_1.2-PV mice spent struggling, swimming and immobile in the FST. **b** Time spent struggling, swimming and immobile in the FST comparing Ca_v_1.2-PV and Ca_v_1.2-Nex mice. ^#^*p* < 0.05, Tukey post hoc test. The p values are from student’s t test (***p < 0.001) and two-way ANOVAs with Tukey’s *post hoc* test, ^#^*p* < 0.05, data are presented as mean ± S.E.M. **(a** Ctrl^PV^: *n* = 16, CKO^PV^: *n* = 19; **b** Ctrl^PV^: *n* = 5, CKO^PV^: *n* = 5; Ctrl^Nex^: *n* = 6, CKO^Nex^: *n* = 6).

Previous studies revealed that Ca_v_1.2 inactivation in glutamatergic neurons enhances active stress-coping behavior^32,33^. Therefore, we tested Ca_v_1.2-PV mice also in the forced swim test (FST). CKO^PV^ mice displayed a more passive stress-coping strategy, characterized by reduced swimming and increased immobility, compared to Ctrl^PV^ littermates (Student’s t test, struggling time: t_33_ = 0.2074, *p* = 0.8370; swimming time: t_33_ = 4.013, *p* = 0.0003; time immobile: t_33_ = 4.362, *p* = 0.0001) (Fig. 4a). In light of previous results, we performed another FST to directly compare the behavior of Ca_v_1.2-PV mice with Ca_v_1.2-Nex mice, the latter lacking *Cacna1c* expression in forebrain glutamatergic neurons. We analyzed the data using two-way ANOVA with genotype and line as factors to assess overall effects, and we compared CKO mice to their respective Ctrl littermates using multiple comparisons test. We confirmed the passive stress-coping behavior of CKO^PV^ mice in the independent cohort of Ca_v_1.2-PV mice (Fig. 4b). In contrast, CKO^Nex^ mice showed enhanced active stress-coping behavior compared to respective Crtl^Nex^ littermates thereby confirming previous findings (two-way ANOVA; struggling time: interaction, F_(1, 18)_ = 0.3447, *p* = 0.5644; genotype, F_(1, 18)_ = 1.747, *p* = 0.2028; mouse line, F_(1, 18)_ = 0.8550, *p* = 0.3674; swimming time: interaction, F_(1, 18)_ = 17.47, *p* = 0.0006; genotype, F_(1, 18)_ = 0.04459, *p* = 0.8351; mouse line, F_(1, 18)_ = 0.1666, *p* = 0.6879; time immobile: interaction, F_(1, 18)_ = 16.01, *p* = 0.0008; genotype, F_(1, 18)_ = 0.02094, *p* = 0.8865; mouse line, F_(1, 18)_ = 0.07356, *p* = 0.7893) (Fig. 4b). Taken together, the conditional inactivation of *Cacna1c* revealed a cell type-specific bidirectional effect of Ca_v_1.2 deletion on stress-coping behavior.

**Fig. 5:**
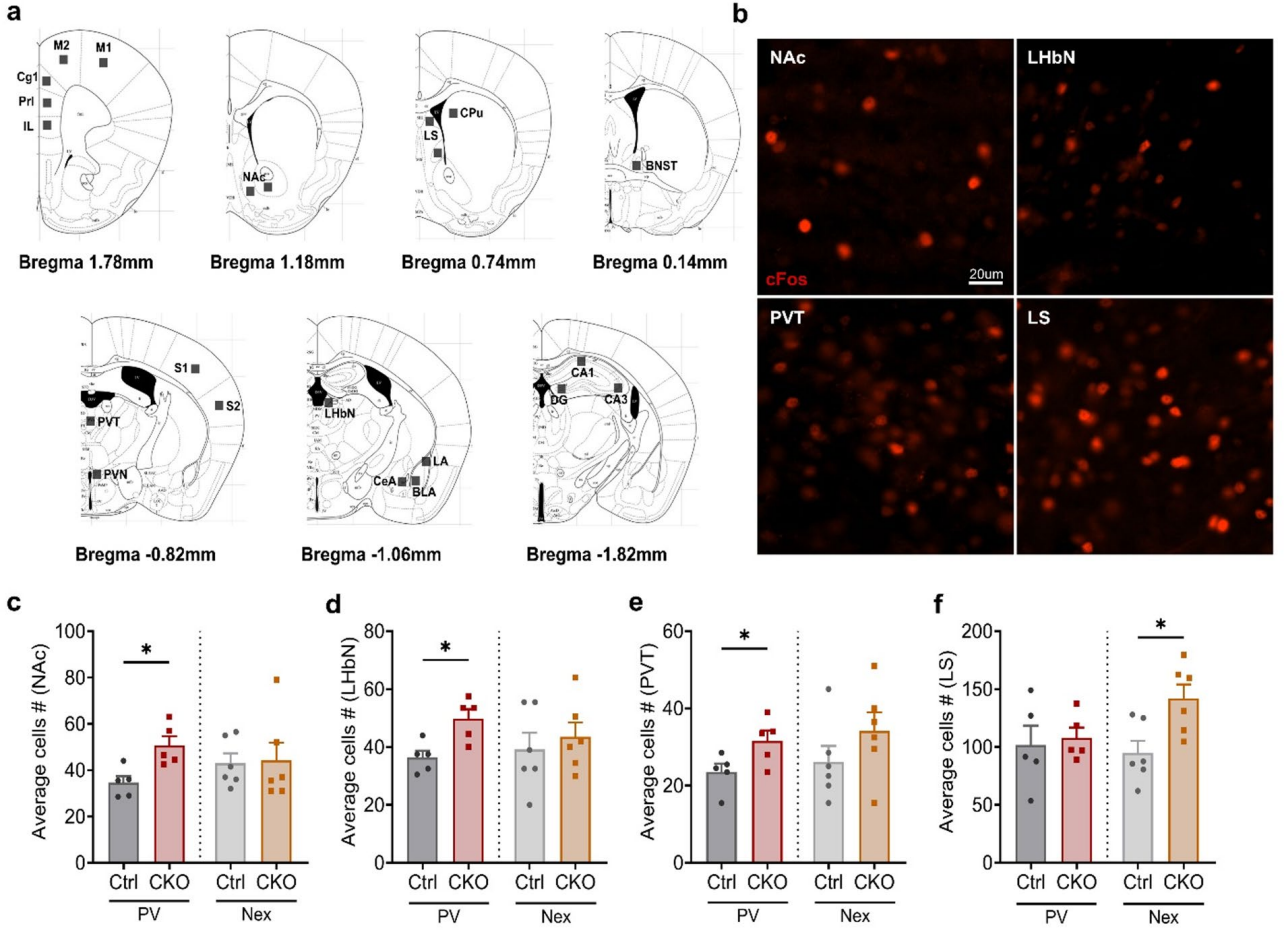
Loss of Ca_v_1.2 in PV^+^ or excitatory neurons activates distinct stress-responsive brain regions. **a** Stress responsive brain regions selected for cFos quantification. For abbreviations see Supplementary Fig. 1. **b** Representative images of cFos activation in Ca_v_1.2-PV (nucleus accumbens, NAc; lateral habenular nucleus, LHbN; paraventricular thalamic nucleus, PVT) and Ca_v_1.2-Nex (lateral septum, LS) mice. Altered cFos expression in **c** NAc, **d** LHbN, **e** PVT in Ca_v_1.2-PV mice and in **f** LS in Ca_v_1.2-Nex mice. Scale bar: 20 μm. The p values are from student’s t test, ^*^p < 0.05, data are presented as mean ± S.E.M. (**c**–**e** Ctrl^PV^: *n* = 5, CKO^PV^: *n* = 5; **f** Ctrl^Nex^: *n* = 6, CKO^Nex^: *n* = 6).

To identify the underlying brain regions and circuits potentially driving this bidirectional response, we quantified the expression of the immediate early gene cFos in brain regions implicated in the stress response 90 min after the FST (Fig. 5a). In the majority of brain regions, we did not detect significant changes in FST-induced cFos expression when comparing CKO and respective Ctrl littermates of the two mouse lines (Supplementary Fig. 1). In CKO^PV^ mice, we observed increased cFos expression in the nucleus accumbens (NAc) (Student’s t test; t_8_ = 3.307, *p* = 0.0107), lateral habenular nucleus (LHbN) (Student’s t test; t_8_ = 3.345, *p* = 0.0102) and paraventricular thalamic nucleus (PVT) (Student’s t test; t_8_ = 2.355, *p* = 0.0464) compared to the respective Ctrl^PV^ mice, reflecting heightened neuronal activity in these regions (Fig. 5b-e). In contrast, CKO^Nex^ mice showed significantly enhanced cFos expression only in the lateral septum (LS) compared to respective Ctrl^Nex^ mice (Student’s t test; t_10_ = 2.916, *p* = 0.0154) (Fig. 5b, f). In sum, these results highlight the cell type-dependent, bidirectional role of Ca_v_1.2 in stress response regulation, with PV^+^ neuron-specific Ca_v_1.2 loss inducing passive stress-coping involving increased activation of neurons in the NAc, LHbN and PVT, while inactivation restricted to forebrain glutamatergic neurons promotes active stress-coping accompanied by enhanced LS activity. Together, these findings underscore the importance of cell type-specific Ca_v_1.2-mediated signaling in orchestrating stress-coping strategies and regulating brain circuit activity.

## Discussion

Dysfunction or loss of PV^+^ neurons is increasingly recognized as a contributing factor in the pathogenesis of psychiatric disorders^31,34,35^. Given that L-type calcium channels in PV^+^ neurons have been shown to regulate parvalbumin expression and interneuron development in vitro ^28,29,36,37^, we generated a novel mouse model with Ca_v_1.2 specifically deleted from PV^+^ neurons during development. We subjected CKO^PV^ and Ctrl^PV^ mice to a behavioral test battery assessing endophenotypes relevant to psychiatric disorders. Our comprehensive investigations revealed that the PV^+^ neuron-specific Ca_v_1.2 deletion has an anxiogenic effect and promotes enhanced passive stress-coping behavior, while general locomotion, social behavior and cognitive performance are unaffected. Additionally, we demonstrated that the stress-coping behavior in the FST is bidirectionally altered depending on whether Ca_v_1.2 is removed from PV^+^ or forebrain glutamatergic neurons, resulting in more passive or active coping behavior, respectively. This cell type-specific behavioral effect was associated with the differential activation of stress-responsive brain regions providing insights into potential circuits and mechanisms underlying the opposing stress-coping behaviors observed in Ca_v_1.2-PV compared to Ca_v_1.2-Nex mice.

Using RiboTag mice as a proxy to report the recombination efficiency of the PV-Cre line, we observed that the HA staining perfectly matched the previously reported endogenous PV expression pattern. The RiboTag reporter has been widely used to assess Cre-dependent recombination and cell type-specific gene expression across diverse cell populations, demonstrating high sensitivity and reliability as indicators of recombined cell populations^38–40^. In addition, the Cre-mediated activation of RPL22-HA confirmed previous reports on the Cre activity of the PV-Cre line including the recombination in cerebellar excitatory granule cells originating from transient Cre expression during development^41^.

Loss of Ca_v_1.2 channels in PV^+^ inhibitory neurons led to increased anxiety-related behavior without affecting basal exploratory activity. Interestingly, cell type-specific inactivation of Ca_v_1.2, as opposed to pan-neuronal deletion, appears to induce anxiety more robustly while leaving locomotor activity unaffected, suggesting a distinct cell type-specific role for Ca_v_1.2 in modulating anxiety^32,33,42–44^. This highlights the importance of Ca_v_1.2 channels in regulating PV^+^ neuron functionality and their downstream effects on anxiety-modulating circuits.

The loss of PV^+^ neurons has been linked to ASD-like behavioral impairments particularly involving social deficits^31^. Accordingly, glutamatergic neuron-specific Ca_v_1.2 knockout mice showed impaired social behavior^32,45^. However, our study shows that CKO^PV^ mice exhibit normal social preference and social memory retrieval which further emphasizes the cell type-specific roles of Ca_v_1.2 in modulating social behavior. Seemingly, calcium influx through Ca_v_1.2 channels in PV^+^ neurons does not have a direct impact on the modulation of social behaviors but instead, primarily affects anxiety and emotional processes. This distinction underscores the nuanced role of Ca_v_1.2 in PV^+^ neurons, where its contribution may vary depending on the specific behavioral domain and neural circuit involved. Further studies are warranted to investigate how Ca_v_1.2 influences the molecular and synaptic mechanisms underlying PV^+^ neuron function in different behavioral contexts.

Furthermore, we report comparable performance of Ctrl^PV^ and CKO^PV^ mice in the Y maze, NOR, SOR and MWM which suggests that hippocampus-dependent cognitive function was unaffected by Ca_v_1.2 deficiency in PV^+^ neurons. Considering their functional redundancy, it is likely that an upregulation of Ca_v_1.3 in hippocampal PV^+^ neurons compensates for the loss of Ca_v_1.2, thus enabling normal hippocampus-dependent cognitive performance. Indeed, such an enhanced expression of Ca_v_1.3 in the absence of Ca_v_1.2 during early embryogenesis has been reported in cardiomyocytes and murine embryonic hearts, suggesting that Ca_v_1.3 channels can compensate for loss of Ca_v_1.2 during development^46,47^.

PV^+^ neurons are critical regulators of stress responses and their dysfunction can result in stress-related phenotypes^16,48^. In this study, CKO^PV^ mice exhibited a passive stress-coping strategy in the FST, characterized by increased immobility and a complementary reduction of swimming time. Similarly, a previous study showed that Ca_v_1.2 deficiency in serotonin-expressing neurons also results in a passive stress-coping response^49^. In contrast, we previously reported and confirmed in this study that Ca_v_1.2 deficiency in forebrain glutamatergic neurons leads to more active stress-coping, indicating a cell type-specific divergence in the stress responses driven by Ca_v_1.2 function^32^. Of note, our results also underscore the importance of using littermates as appropriate controls. Ctrl^PV^ and Ctrl^Nex^ mice significantly deviate in their behaviors in the FST which might be related to difference in their genetic background introduced by breeding to PV- and Nex-Cre mouse lines, respectively.

PV^+^ neurons are essential for maintaining the E/I balance within neural circuits, and respective disturbances are a hallmark of several psychiatric conditions. It is plausible that Ca_v_1.2 loss in specific neuronal populations, whether excitatory or inhibitory, leads to a shift in E/I balance, thus resulting in opposing maladaptive stress responses. Indeed, a comparable study that investigated the role of AHNAK, a regulator of L-type VGCCs, in glutamatergic and PV^+^ neurons reported a similar opposing effect on the stress response^50^. Another consequence of increased or decreased E/I ratio is a possible hyperactivation of stress response-related circuits and brain regions. Supporting this hypothesis, cFos quantification of FST-activated brain regions revealed an increased activity in the NAc, LHbN, and PVT in CKO^PV^ mice, while CKO^Nex^ mice exhibited hyperactivity in the LS. Each of these regions is involved in the regulation of stress-related behaviors, however, the specific role of Ca_v_1.2 in PV^+^ and glutamatergic neurons within these regions, as well as the circuit dynamics underlying these distinct stress responses require further investigation^51–55^.

A limitation of the present study is that all experiments were conducted exclusively in male mice. Future studies will be necessary to extend these analyses to sex-specific effects of Ca_v_1.2 deletion in PV^+^ neurons and their contribution to behavioral phenotypes relevant to psychiatric disorders. These studies will have to closely monitor estrogen levels during the estrus cycle which have been shown to affect PV^+^ neuron excitability^56^.

In conclusion, we demonstrate that Ca_v_1.2 deficiency in PV^+^ neurons significantly alters behavioral and circuit-level responses, emphasizing the cell type-specific roles of Ca_v_1.2 in emotional behaviors and stress regulation. While prior studies have implicated PV^+^ neurons and Ca_v_1.2 as individual factors in psychiatric disorders, this study is, to our knowledge, the first to specifically explore the role of Ca_v_1.2 in PV^+^ neurons in modulating behaviors related to psychiatric disorders. Moreover, we revealed a striking cell type-dependent divergence in the stress responses and of concomitantly activated brain regions, depending on whether Ca_v_1.2 is inactivated in forebrain glutamatergic or PV^+^ neurons. These findings highlight the distinct yet complementary contributions of PV^+^ and glutamatergic circuits to stress adaptation. They also provide critical insights into the cellular and network mechanisms underlying stress regulation and coping strategies, and potential implications for targeted therapies in psychiatric disorders.

## Materials and methods

### Analysis of single-cell sequencing dataset

Single-cell RNA sequencing data from the mouse brain was obtained from a publicly available dataset^57^. The dataset was accessed via the Allen Brain Cell Atlas, which provides a repository for data download, documentation, and a Jupyter Notebook for data retrieval and analysis. Data preprocessing was performed in Jupyter Notebook with Python using the Pandas and Scanpy libraries. Cells expressing *Cacna1c* were identified based on nonzero expression levels of the gene. *Cacna1c* expression across neurotransmitter-defined cell groups and GABAergic subpopulations was visualized in combined bar–violin plots in Jupyter Notebook. Cells were categorized by their annotated neurotransmitter class. GABA cells were extracted based on neurotransmitter annotation, and subgroups were defined by the presence of key marker genes, including Gad1, Gad2, Slc32a1, Sst, Vip, and Pvalb. Cell counts were displayed using a bar plot with a split y-axis to accommodate different group sizes, and expression distributions were overlaid as violin plots on a secondary axis.

### Mice

The Ca_v_1.2-PV mouse line, in which *Cacna1c* is selectively deleted in parvalbumin-expressing (PV^+^) neurons, was generated by crossing *Cacna1c*^*lox/lox*^ mice (*Cacna1c*^*tm3Hfm*^) with *PV-Cre* (*Pvalb*^*tm1(cre)Arbr*^, JAX strain #:017320) and *RiboTag* mice (*Rpl22*^*tm1.1Psam*^, JAX strain #:029977)^58–60^. *RiboTag* mice express a Cre recombinase-dependent hemagglutinin epitope-tagged (HA-Tag) ribosomal protein L22 (Rpl22) which was used as a proxy to evaluate the efficiency and spatial pattern of Cre-mediated recombination. Intercrossing of the three starter lines eventually resulted in control (Ctrl^PV^; *Cacna1c*^*lox/lox*^::*Rpl22*^*HA/HA*^) and conditional knockout (CKO^PV^; *Cacna1c*^*lox/lox*^::*Rpl22*^*HA/HA*^::*PV-Cre*) mice. *Ca*_*v*_*1.2-Nex* control (Ctrl^Nex^; *Cacna1c*^*lox/lox*^*)* and conditional knockout (CKO^Nex^; *Cacna1c*^*lox/lox*^::*Nex-Cre*) mice were generated as previously described^32^. Transgenic mice were bred in specific pathogen free conditions at the central animal facility at the Max Planck Institute for Biochemistry (Martinsried, Germany). Mice were group-housed under standard laboratory conditions (22 ± 1 °C, 55 ± 5% humidity) on a 12-hour light-dark cycle with *ad libitum* access to food and water. It has been previously demonstrated that estrogen modulates PV^+^ interneuron excitability via estrogen receptor β ^56^. Therefore, we focused in our experiments on male mice to investigate genotype-dependent differences avoiding confounds resulting from variable estrogen levels during the estrus cycle. A first cohort of male Ca_v_1.2-PV mice was subjected to the complete behavioral test battery to assess locomotion, anxiety-related and social behavior, learning and memory as well as stress-coping behavior (Fig. 1e). An independent cohort of male Ca_v_1.2-Nex and Ca_v_1.2-PV mice was subjected to the FST and used for cFos staining.

### Behavioral analysis

Behavioral characterization of Ca_v_1.2-PV mice was conducted between 9:00 am and 3:00 pm. All tests were performed on 4-6-month-old male mice by an experienced, blinded researcher, following established protocols. Unless otherwise stated, behavioral data were recorded and analyzed using ANY-Maze software (Stoelting Co., Wood Dale, Illinois, USA).

### Exploratory and anxiety-related behaviors

#### Open field test (OFT)

Exploratory behavior in a novel environment was investigated in the OFT. Mice were placed in a corner of a dimly lit (< 30 lx) open field arena (50 cm × 50 cm × 40 cm) and allowed to freely explore the apparatus for 30 min. Total distance traveled, time spent in the inner zone, and number of inner zone entries were measured using the ANY-maze software.

#### Light/dark box (LDB)

The LDB was conducted in a rectangular apparatus divided into a smaller dark zone (15 cm × 25 cm) and a larger illuminated zone (30 cm × 25 cm) connected by a small opening (5 cm × 7 cm). The lit zone was illuminated to 700 lx to create a highly aversive environment. Mice were initially placed in the dark zone and allowed to freely explore the apparatus for 10 min. Time spent in the lit zone and number of entries into the lit zone were scored manually from video recordings of the test.

#### Elevated plus maze (EPM)

The EPM was performed in a plus-shaped elevated maze (30 cm) with two opposite open arms (5 × 30 cm), two closed arms (30 × 5 × 15 cm) and a central zone (5 × 5 cm) was used. The illumination in the open arms was 30 lx and < 10 lx in the closed arms. Mice were placed in the central zone facing one of the closed arms and were allowed to freely explore the maze for 10 min. The time spent in the open arms and number of open arm entries were recorded using the ANY-maze software.

### Cognitive and social behaviors

#### Three-chambered social interaction test (3CT)

Social preference and social novelty memory were assessed using a 3-chambered apparatus consisting of two side chambers (19 cm × 25 cm) containing removable wire cups separated by a central chamber (12 cm × 25 cm). The test was conducted in 3 stages. First stage: Test mouse was placed in the central chamber and allowed to acclimatize to the apparatus and the wire cups for 10 min. Second stage: A male, age-matched conspecific mouse (S1) was placed in one of the wire cups, and the test mouse was allowed to explore for 10 min. Third stage: A novel, male, age-matched conspecific (S2) was placed in the other wire cup and the test mouse was allowed to explore for an additional 10 min. The time spent in each of the 3 chambers (first stage) and interactions with conspecifics were scored manually from video recordings and social preference and social novelty were calculated accordingly.

#### Y-maze spontaneous alternations test

Working memory was assessed using the Y-maze spontaneous alternation task. The apparatus consisted of three opaque arms at 120° angle from each other (30 cm × 10 cm × 15 cm). Illumination in the testing apparatus was maintained at 30 lx. Mice were allowed to freely explore the apparatus for 10 min. The number of triads (for example: ABC, BCA, CAB…) and the total number of arm entries were scored manually, and the percentage of spontaneous alternations was calculated accordingly.

#### Novel object recognition (NOR) test

Short-term recognition memory was assessed using the NOR test in the Y-maze apparatus. Mice were habituated to the apparatus for 10 min, 24 h prior to the testing session. On the day of the experiment, mice were allowed to freely explore two identical objects placed at the end of arms A and B for 15 min. After a 20 min intertrial interval, one object was randomly replaced with a novel object and mice were allowed to explore the objects freely for 5 min. Object interaction times were scored manually and novelty preference, and discrimination index were calculated accordingly.

#### Spatial object recognition (SOR) test

The SOR test was performed in the Y-maze apparatus. The test consisted of three stages: habituation (10 min), acquisition (15 min) and test (5 min) with an ITI of 20 min between the acquisition and test stages. Two identically assembled lego blocks were placed at the end of arms A and B during the acquisition stage and cues of different shapes were used to help the animals orient themselves. During the testing stage of the SOR, the object from either arm A or arm B was moved to arm C randomly. The object interaction times were scored manually and novelty preference, and discrimination index were calculated accordingly.

#### Morris water maze (MWM)

Spatial memory was assessed using the classical MWM. Mice were trained to locate a submerged platform over 5 consecutive days (4 trials per day, each lasting 90 s with a 20-min intertrial interval). A probe trial (without the platform) was conducted 24 h after the last trial of the final training day. The latency to reach the platform and time spent in each quadrant was recorded using ANY-maze software. Average latencies during training and time spent in the target (platform) quadrant during the probe trial were calculated accordingly.

### Stress-coping behavior

#### Forced swim test (FST)

Active versus passive stress-coping behavior was assessed using the FST. The test was performed using a glass beaker (radius 11 cm, height 23.5 cm) filled with 1.5 l water (25 °C). Mice were subjected to a 6-minute test, during which the time spent struggling, swimming, and floating was scored manually.

#### Antibody staining

Mice were sacrificed by an overdose of isoflurane and transcardially perfused with 1× phosphate-buffered saline (PBS) followed by 4% paraformaldehyde (PFA). For cFOS quantification, brain tissue was collected 90 min after the FST. Brains were post-fixed in 4% PFA overnight at 4 °C, cryoprotected in 30% sucrose in PBS and then coronally sectioned at 50 μm thickness. For immunofluorescence staining, brain sections were rinsed in PBS, blocked in blocking solution (1.5% NGS, 0.3% Triton X-100 and 1× PBS) for 1 h at room temperature (RT). The blocking solution was also used for antibody dilution. Sections were incubated with a primary rabbit antibody against cFos (1:1000, Abcam, #ab190289) or the HA-tag (1:500, Cell Signaling Technologies, #C29F4,) at 4 °C overnight. After rinsing, sections were incubated in secondary antibody (1:5000, Thermo Fischer Scientific, goat anti-rabbit Alexa Fluor 568, or AlexaFluor 488) for 2 h at RT. Sections were rinsed, mounted with Fluoromount-G containing DAPI (Southern Biotech, #0100 − 20) and left to dry overnight at RT.

### Image acquisition and cFos quantification

20 brain regions potentially activated during the FST according to previously reported studies, were selected based on the Paxinos and Franklin’s mouse brain atlas (1997) (Fig. 5a). Images were acquired using Olympus SlideScanner VS120S6. After obtaining an overview of each slide to identify each brain region, 200 μm × 200 μm ROIs were drawn across selected brain regions and Z stack images of these areas were taken at 20× magnification with a Z distance of 20 μm and Z spacing of 1 μm. Images were taken from two consecutive sections and image processing and analysis was done using ImageJ. For pre-processing of images, BioFormats plugin (https://www.openmicroscopy.org/bio-formats/downloads/) was downloaded to open .vsi files from the slide scanner. cFos-positive cells were quantified on 8-bit maximum intensity projection images obtained in ImageJ. The images were thresholded, watershed to separate overlapping cells, and automatically quantified using “analyze particles” function. In cases of high background which fell within threshold range, the cells were counted manually.

### Statistical analysis

Statistical analyses were performed using GraphPad Prism v10.0 (GraphPad Software, La Jolla, CA, USA). The sample size was determined to achieve a type 1 error of 0.05 and a type 2 error of 0.2, with an effect size of at least 1.2-fold the pooled standard deviation. Normality was assessed using the Shapiro-Wilk test. Student’s t test (two-tailed) was used to compare behavioral phenotypes of Ca_v_1.2-PV mice. For time-dependent measures, a two-way analysis of variance (ANOVA) with repeated measures was used. Whenever significant main or interaction effects were identified, Bonferroni *post hoc* tests were applied to determine simple effects. For behavioral experiments involving multiple factors (FST across transgenic lines), data were additionally analyzed using two-way ANOVA with genotype and line as factors and Tukey *post hoc* test. For cFos quantification, within-line comparisons were performed using Student’s t test to assess genotype-dependent effects relative to its matched control. This approach was chosen to avoid assumptions of shared baseline across the two transgenic lines and focus only on genotype effects within each cohort. Data are presented as mean ± S.E.M. Statistical significance was defined as *p* < 0.05. Outliers were detected using Grubbs’ test.

## Supplementary Information

Below is the link to the electronic supplementary material.

Supplementary Material 1

## Data Availability

All processed data are in the figures of the manuscript. All raw data are available from the corresponding authors upon request.
