# Supplementary material for: Cacna1c deficiency in parvalbumin-expressing neurons promotes anxiety and passive stress-coping behavior
Source: Sci Rep. 2026 Apr 20;16:12870. doi: 10.1038/s41598-026-48841-4 (PMC13096310; doi:10.1038/s41598-026-48841-4)
Supplement: Supplementary file 1 — Supplementary Material 1 [file 41598_2026_48841_MOESM1_ESM.pdf]

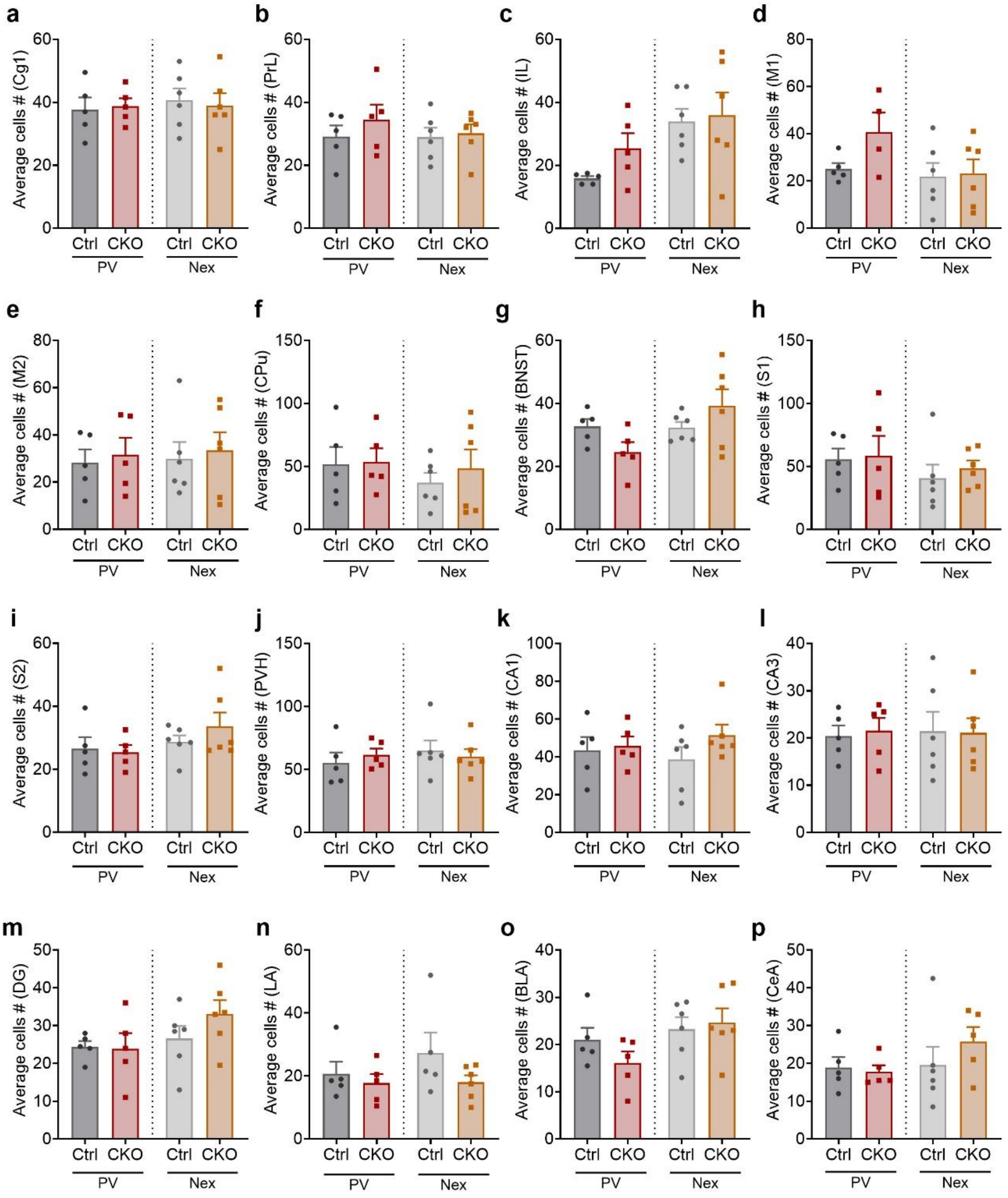

Supplementary Figure 1. cFos quantification in stress responsive brain regions in $Ca_V1.2$-PV and $Ca_V1.2$-Nex mice. Data presented as mean ± S.E.M. [(a-p) PV: Ctrl n = 5, CKO n = 5; Nex: Ctrl n = 6, CKO n = 6)]. Cg1 – cingulate cortex, PrL – prelimbic cortex, IL – infralimbic cortex, M1 – primary motor cortex, M2 – secondary motor cortex, CPu – caudate putamen, BNST – bed nucleus of the stria terminalis, S1 – primary somatosensory cortex, S2 – secondary somatosensory cortex, PVH – paraventricular nucleus of hypothalamus, CA1 – cornu ammonis 1, CA3 – cornu ammonis 3, DG – dentate gyrus, LA – lateral amygdala, BLA – basolateral amygdala, CeA – central amygdala.
